# The Nutmeg Lung Pattern in a Fetus with Hypoplastic Left Heart Syndrome and Turner Syndrome

**DOI:** 10.1007/s00246-025-03873-x

**Published:** 2025-04-27

**Authors:** Katrin Fricke, Katarina Övermo Tydén, Gunnar Bergman, Erik Hedström

**Affiliations:** 1 Pediatric Cardiology, Pediatric Heart Center, Skåne University Hospital, Lund, Sweden; 2 Pediatrics, Department of Clinical Sciences Lund, Lund University, Lund, Sweden; 3 Department of Pediatric Cardiology, Karolinska University Hospital, Stockholm, Sweden; 4 Department of Women’s and Children’s Health, Karolinska Institutet, Stockholm, Sweden; 5 Clinical Physiology, Department of Clinical Sciences Lund, Lund University, Lund, Sweden; 6 Department of Clinical Physiology, Skåne University Hospital, Lund, Sweden; 7 Diagnostic Radiology, Department of Clinical Sciences Lund, Lund University, Lund, Sweden; 8 Department of Radiology, Skåne University Hospital, Lund, Sweden

**Keywords:** Nutmeg lung pattern, Fetal cardiac MRI, Turner syndrome, Hypoplastic left heart syndrome

## Abstract

The “nutmeg lung pattern” on fetal magnetic resonance imaging (MRI) indicates pulmonary lymphangiectasia. This is associated with adverse outcomes, particularly in fetuses with congenital heart defects and impaired pulmonary venous return. Whereas lymphedema is common in fetuses with Turner syndrome, pulmonary lymphangiectasia is not. A 26-year-old woman presented with a fetus with hypoplastic left heart syndrome (HLHS) without restrictive atrial septum (RAS). The family declined amniocentesis, yet non-invasive prenatal testing showed an increased risk for Turner syndrome. The patient underwent a fetal MRI as part of a blinded research protocol. Postnatal echocardiogram confirmed the fetal echocardiographic findings without evidence of RAS. Norwood stage I palliation was performed at two days of age. Significant neonatal respiratory morbidity including pneumonia, diaphragmatic and vocal cord pareses and chylothorax occurred. Subsequent review of fetal MRI revealed a prominent thoracic duct and mild pulmonary lymphangiectasia. Turner syndrome was confirmed by genetic testing. After one month, the patient was discharged to her home hospital with respiratory support, which was discontinued a few weeks later. Respiratory problems continued, but the vocal cord paresis resolved over time. This is a unique case of a fetus with HLHS/non-RAS with mild fetal pulmonary lymphangiectasia, and significant neonatal respiratory morbidity, probably in part due to Turner syndrome. The infant survived the neonatal period and underwent uneventful Glenn surgery. The patient’s tolerance to the total cavopulmonary connection (TCPC) procedure is yet to be seen. An MRI lymphography should precede it to assess residual lymphatic abnormalities and serve as baseline for post-TCPC changes.

## Introduction

Pulmonary lymphangiectasia can be visualized on fetal magnetic resonance imaging (MRI) and is referred to as the “nutmeg lung pattern” [1–3]. In these fetuses, lymphatic channels are seen as linear tubular structures radiating from the hilae toward the pleural surface [1]. This pattern is associated with high neonatal mortality rates, particularly in fetuses with congenital heart defects (CHD) [2, 3]. In the CHD subgroup with impaired pulmonary venous return, such as hypoplastic left heart syndrome (HLHS) with restrictive or intact atrial septum (RAS/IAS) or obstructed total pulmonary venous return (TAPVR), increased pulmonary venous pressure leads to changes in the pulmonary vasculature and dilated lymphatic channels with increased lymphatic drainage [3]. However, primary pulmonary lymphangiectasia, an innate lymphatic anomaly, may also occur in isolated cases and may have a more favorable outcome [3].

In fetuses with Turner syndrome, the fetal ultrasound findings due to lymphatic abnormalities are diffuse fetal edema with increased nuchal translucency and fetal hydrops [4] or, less commonly, fetal chylosis [5]. However, pulmonary lymphangiectasia visualized as the nutmeg lung pattern on fetal MRI has not been described in fetuses with Turner syndrome.

## Case Presentation

### Fetal Course

**Fig. 1:**
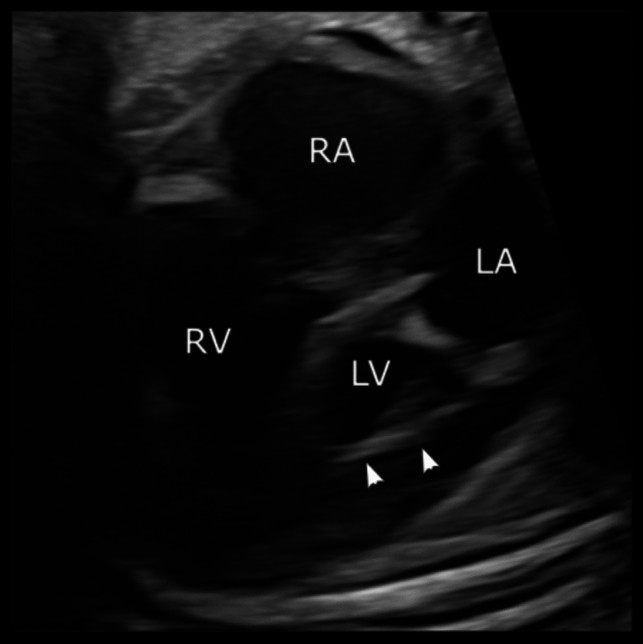
Fetal echocardiogram at gestational week 38 + 2 in the four-chamber view. Hypoplastic left heart syndrome with a globular left ventricle and endocardial fibroelastosis (arrowheads) are shown. *LV* left ventricle, *RV* right ventricle, *LA* left atrium, *RA* right atrium

**Fig. 2:**
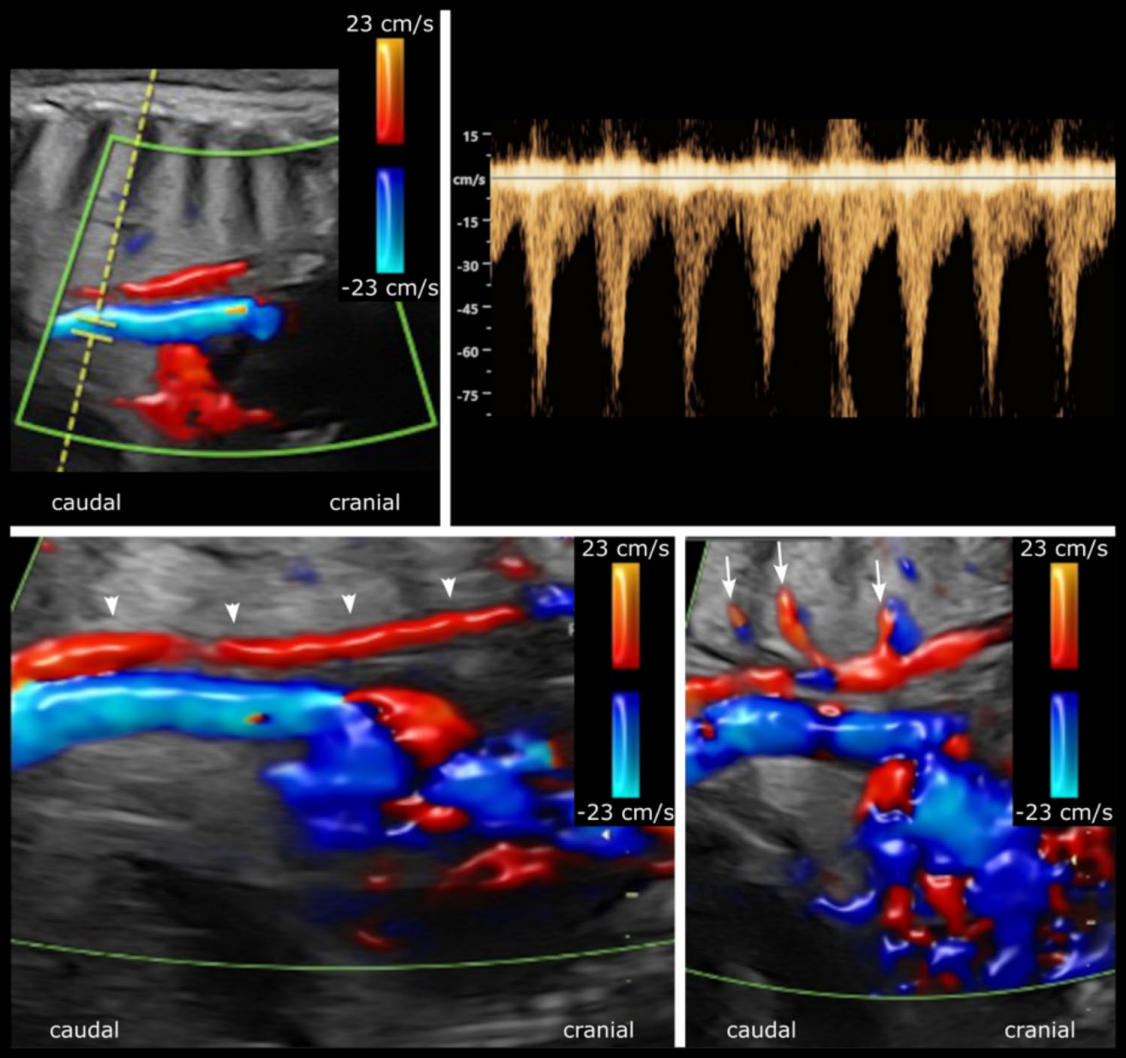
Fetal echocardiogram at gestational week 38 + 2 in the sagittal view. Two tubular structures with flow in opposite directions are shown (top left). The probe position is such that the vessel with flow in the caudal direction is blue, but shows pulsatile flow corresponding to the descending aorta, whereas the tubular structure posteriorly and to the right of the descending aorta shows slow flow (red) in the cranial direction. The Doppler curve for the pulsed flow marker in the descending aorta is shown (top right). The tubular structure posteriorly and to the right of the descending aorta was initially suspected to be a dilated azygos vein, considering its location, diameter, and flow direction. In retrospect, after reviewing the fetal MRI along with the postnatal echo and CT, the differential diagnosis was instead suggested to be that of a dilated thoracic duct (bottom left; arrowheads) with dilated lymphatic channels (bottom right: arrows)

A 26-year-old pregnant woman with no family history of CHD and low risk for genetic abnormalities presented for her first trimester screening ultrasound, which resulted in the diagnosis of HLHS. Amniocentesis was not desired by the family, yet non-invasive prenatal testing showed an increased risk for Turner syndrome. The last fetal echocardiogram at 38 + 2 weeks of gestation showed an HLHS variant with aortic and mitral valve stenosis (AS-MS), a globular left ventricle with endocardial fibroelastosis and normal right ventricular function without significant tricuspid regurgitation (Fig. 1). There was no evidence of a restrictive atrial septum (RAS) with abundant left-to-right shunting and with normal flow profiles in non-dilated pulmonary veins. No hydrops, pleural effusions, or situs abnormalities were observed with clearly visible and normally connected superior and inferior venae cavae. However, a dilated vascular structure was noted posteriorly and to the right of the aorta, suggesting the presence of a dilated azygos vein (Fig. 2).

Being considered to be in the standard risk group, vaginal delivery at term was planned for at our tertiary center without the catheterization lab on standby. Prior to delivery, the patient underwent fetal cardiovascular MRI as part of a blinded research protocol.

### Delivery and Neonatal Outcome

The infant was born by spontaneous vaginal delivery at 38 + 4 weeks of gestation. The newborn presented with an APGAR of 9–9–9, a birth weight of 3636 g, and a height of 51 cm. The initial preductal saturation was 93%. Postnatal genetic testing later confirmed Turner syndrome.

**Fig. 3:**
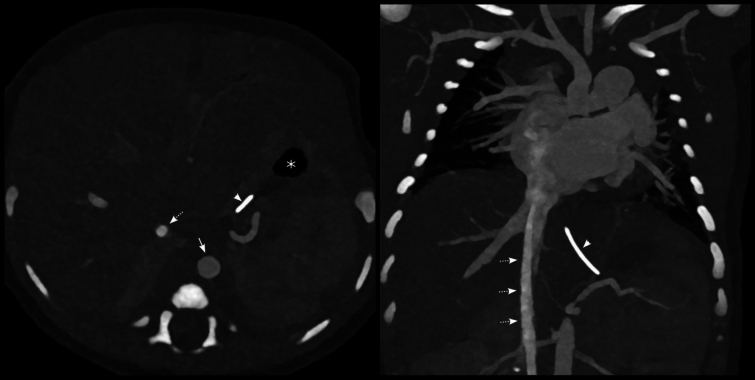
Computed tomography within the first 24 h after birth with contrast agent administered through the dorsal foot vein. Situs solitus with normally connected superior and inferior venae cavae and no presence of an azygos continuation were confirmed. *White dashed arrows*, inferior vena cava; *white arrow*, abdominal aorta; *white arrowheads*, nasogastric tube pointing toward the patient’s left side and stomach; *white asterisk*, stomach on the patient’s left side

The postnatal echocardiogram and computed tomography (CT) at one day of age confirmed the fetal echocardiographic findings of the anatomic HLHS subtype AS-MS with a globular left ventricle and endocardial fibroelastosis. There was no clinical or echocardiographic evidence of RAS. Situs solitus with normally connected superior and inferior venae cavae and no azygos continuation were also confirmed by the postnatal echocardiogram and CT (Fig. 3). Thus, the tubular structure with Doppler flow seen on the fetal echo, located posteriorly and to the right of the aorta with flow toward the upper half of the body, was now less likely to be a dilated azygos vein as suspected during the fetal echo.

The initial chest x-ray showed a normal cardiac silhouette and hilar regions, and no significant pulmonary edema, pleural effusion, or atelectasis. However, soon thereafter clinical signs of pulmonary congestion occurred, related to the large atrial septal defect and patent arterial duct. Continuous positive airway pressure (CPAP) and early Norwood stage I procedure at two days of age were deemed necessary. The neonate was successfully extubated three days later but reintubated due to left-sided pneumonia with extensive consolidation and subsequent identification of extended-spectrum beta-lactamase *E. coli* (ESBL). Simultaneously, left-sided diaphragmatic and vocal cord pareses were diagnosed and ventilator support with prolonged weaning was needed due to decreased saturation and elevated pCO_2_. Although improving at first, acute respiratory distress unexpectedly occurred two and a half weeks after surgery. Chest x-ray and CT revealed significant left-sided pleural effusion requiring drainage for five days, later identified as chylous.

**Fig. 4:**
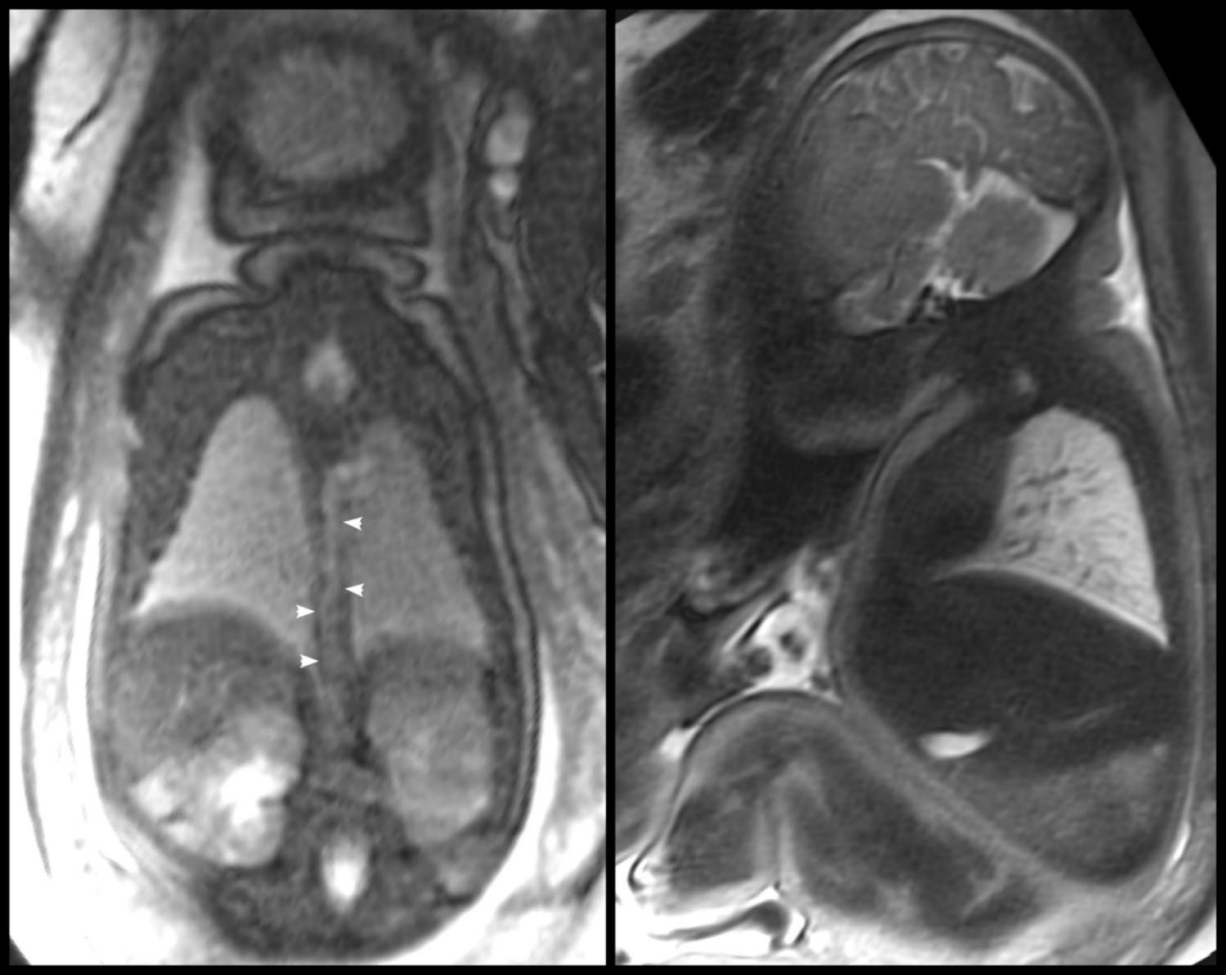
Fetal magnetic resonance imaging at 38 + 3 gestational weeks. A prominent thoracic duct passing from the patient’s right side to the left at the normal thoracic vertebral level (left; white arrowheads) and slight heterogeneous appearance of the pulmonary parenchyma with bright linear structures indicative of mild pulmonary lymphangiectasia are shown (right)

Subsequent review of the initially blinded fetal cardiovascular MRI scan revealed a prominent thoracic duct and pulmonary changes consistent with mild pulmonary lymphangiectasia (Fig. 4). A dilated azygos vein could not be shown.

After one month of inpatient care at our tertiary center, the patient was discharged to her home hospital with ventilatory support by high-flow nasal cannula (HFNC) therapy, which was discontinued a few weeks later.

### Clinical Course During Interstage I, Bidirectional Glenn and Interstage II

Respiratory problems continued, including excessive phlegm, asthma-like symptoms, and recurrent pleural effusions, but the vocal cord paralysis resolved over time. Even though the patient initially was dependent on a gastric tube and had poor weight gain, the assistance of a dietician and a special diet solved these issues.

The pre-Glenn CT and the subsequent assessment of the previous CT scans showed the concomitant presence of unobstructed partial anomalous pulmonary venous drainage (PAPVD) from the right superior pulmonary vein to the superior vena cava. This finding led to a combined bidirectional Glenn and PAPVD surgery at seven months of age. Surgery was uneventful and the child is now awaiting completion of a total cavopulmonary connection (TCPC).

## Discussion

This case report presents a fetus with HLHS/non-RAS and Turner syndrome with mild pulmonary lymphangiectasia and a dilated thoracic duct, which was an incidental finding on fetal cardiovascular MRI performed for research purposes and may be explained by the fetus’ genetic abnormality. The newborn survived the neonatal period but had significant pulmonary morbidity, which may be at least partially explained by the underlying genetic syndrome and potentially related to the findings of fetal pulmonary lymphangiectasia, although mild.

### HLHS and Secondary Pulmonary Lymphangiectasia

Fetuses with HLHS and RAS/IAS are particularly at risk for adverse intrauterine and neonatal outcome. The impaired pulmonary venous return due to RAS/IAS leads to increased pulmonary venous pressure with secondary changes in the pulmonary vasculature and dilated lymphatic channels visualized as the nutmeg lung pattern on fetal MRI [1, 3]. The occurrence of this pattern is associated with significant neonatal mortality in patients with HLHS, as reported in the previous studies [2, 3]. In fetuses with HLHS-RAS/IAS, a cesarean section with cath-lab on standby is usually performed, and in fetuses with more severe pulmonary lymphangiectasia, comfort care may be considered already before birth if the changes are known.

Given the fetal and postnatal echocardiographic and clinical presentation of HLHS without RAS/IAS, the blinded research fetal MRI finding of mild pulmonary lymphangiectasia can be seen as surprising. Even though the newborn survived the neonatal period there was, however, significant pulmonary morbidity in the first months of life with recurrent chylosis and the need for prolonged respiratory support.

Lymphatic dysfunction in patients with HLHS/non-RAS may occur at all stages of univentricular palliation, but preferentially after Glenn or, more commonly, after TCPC surgery [6]. In these cases, chylothorax may result from retrograde perfusion of the lymphatic system or early postoperatively from trauma following cardiac surgery [6]. The patient in this report developed chylothorax more than two weeks after Norwood stage I surgery, which thus makes a traumatic genesis less likely. Accordingly, we suspect that factors other than HLHS/non-RAS may have caused the fetal pulmonary changes in this particular case, which could also in part explain the neonatal pulmonary morbidity in the first months of life.

### Turner Syndrome and Primary Pulmonary Lymphangiectasia

Turner syndrome is a genetic disorder with partial or complete absence of one X chromosome. It affects multiple organ systems, including the heart, with a high incidence of left heart lesions including HLHS [7].

Fetuses with Turner syndrome may be affected by impairment of the lymphatic system, resulting in diffuse fetal edema with increased nuchal translucency, fetal chylosis as well as fetal hydrops [4, 5], which may contribute to the high rates of miscarriage previously reported [4, 8, 9]. It should be noted that the fetus in the current report did not have any of the lymphatic anomalies mentioned above, despite the presence of mild pulmonary lymphangiectasia and a dilated thoracic duct, which have not been reported before in fetuses with Turner syndrome. However, in extremely rare cases, the dysfunction of the lymphatic system in patients with Turner syndrome is known to affect other organ systems such as the intestines [10].

Primary pulmonary lymphangiectasia, an innate lymphatic anomaly, may occur because previously formed lymphatic channels have not regressed or because the pulmonary lymphatic channels do not communicate with the systemic lymphatic channels [1, 11]. Syndromic primary pulmonary lymphangiectasia has been reported in fetuses with Down syndrome, Noonan syndrome, and other rare syndromes, but not previously in Turner syndrome [1, 11]. Although the mortality rate related to pulmonary lymphangiectasia has been earlier reported to be almost 100% [11, 12], it tends to be more favorable in more recent studies [3]. Nevertheless, respiratory symptoms such as tachypnea or severe respiratory distress with cyanosis are common in neonates with primary pulmonary lymphangiectasia [11].

The presented patient showed MRI signs of only mild pulmonary lymphangiectasia, which may explain the more favorable neonatal outcome, albeit with significant pulmonary symptoms within the first months of life.

The dilated thoracic duct seen on fetal MRI could be a sign of abnormal lymphatic drainage, which has not been previously described in fetuses with Turner syndrome with or without HLHS. However, in a recent study of patients with Noonan syndrome and clinical signs of lymphatic dysfunction, MR lymphangiography showed partial or complete absence of the thoracic duct and lymphatic reflux in the intercostal, mediastinal, and other lymphatics, the latter similar to the suspected findings in our patient [13]. Anomalies of the thoracic duct are well visualized with postpartum MR lymphography, whereas ultrasound can visualize dilated lymphatic vessels [14, 15]. Fetal studies demonstrating a dilated thoracic duct by fetal MRI or fetal echo are not reported. Taken together, the tubular structure with Doppler flow identified on fetal echo may be a dilated thoracic duct. However, the existence of a dilated azygos vein in the fetus cannot be completely excluded, despite the presence of a normal situs with a normal inferior vena cava on fetal examination, and the lack of its demonstration on postpartum echo and CT.

## Conclusion

This is a unique case of a fetus with Turner syndrome, HLHS/non-RAS, and mild fetal pulmonary lymphangiectasia and a dilated thoracic duct. Neonatal respiratory morbidity with prolonged respiratory support and chylothorax were observed, probably in part due to the underlying genetic disease. The infant survived the neonatal period and underwent uneventful Glenn surgery. It remains to be seen how the patient will cope with TCPC surgery. This should be preceded by MR lymphography to assess any remaining lymphatic abnormalities and to serve as a baseline for evaluating potential changes after TCPC completion.

## Data Availability

No datasets were generated or analysed during the current study.
